# The Possible Effect of the Long-Term Use of Glucagon-like Peptide-1 Receptor Agonists (GLP-1RA) on Hba1c and Lipid Profile in Type 2 Diabetes Mellitus: A Retrospective Study in KAUH, Jeddah, Saudi Arabia

**DOI:** 10.3390/diseases11010050

**Published:** 2023-03-14

**Authors:** Ghada M. A. Ajabnoor, Kamal Talat Hashim, Mohammed Meshari Alzahrani, Abdullah Zeid Alsuheili, Abdullah Fahad Alharbi, Amani Matook Alhozali, Sumia Enani, Basmah Eldakhakhny, Ayman Elsamanoudy

**Affiliations:** 1 Department of Clinical Biochemistry, Faculty of Medicine, King Abdulaziz University, Jeddah 22252, Saudi Arabia; 2 King Abdulaziz University Hospital, Faculty of Medicine, King Abdulaziz University, Jeddah 22252, Saudi Arabia; 3 Faculty of Medicine, King Abdulaziz University, Jeddah 22252, Saudi Arabia; 4 Department of Medicine, College of Medicine, King Abdulaziz University, Jeddah 22252, Saudi Arabia; 5 Department of Food and Nutrition, Faculty of Human Sciences and Design, King Abdulaziz University, Jeddah 21589, Saudi Arabia; 6 Medical Biochemistry and Molecular Biology, Faculty of Medicine, Mansoura University, Mansoura 35516, Egypt

**Keywords:** type 2 diabetes, GLP-1 receptor agonist, dyslipidemia, HbA1c

## Abstract

(1) Background: Type 2 diabetes (T2DM) is a chronic metabolic disease with serious health complications. T2DM is associated with many chronic illnesses, including kidney failure, cardiovascular diseases (CVD), vision loss, and other related diseases. Obesity is one of the major factors associated with insulin resistance and dyslipidemia. Recently, the development of GLP-1 Receptor agonist (GLP-1RA) showed great therapeutic potential for T2DM. Aim: To retrospectively investigate the association of the long-term use of GLP-1RA therapy in T2DM patients with HbA1c levels and dyslipidemia. (2) Methods: Retrospective data collection and analysis of demographic, clinical records, and biochemical parameters were carried out for 72 T2DM taking GLP-1RA treatments for six months. (3) Results: A total of 72 T2DM patients with a mean age = 55 (28 male and 44 female) were divided into two groups. Group 1 received statins (*n* = 63), and group 2 did not receive statins (*n* = 9). The GLP-1RA effect on BMI was significantly decreased in group 1 (*p* < 0.01). A significant effect was observed for HbA1c in both groups for six months of treatment duration (*p* < 0.05). The AST levels significantly decreased in group 2 from 25.2 to 19.4 U\L (*p* = 0.011). (4) Conclusions: GLP-1RA treatments were associated with weight reduction and improved glycemic control for T2DM patients. Moreover, it is suggested that it has anti-inflammatory and hepatoprotective effects. However, no direct association was found with the lipid profile in all groups of T2DM.

## 1. Introduction

Type 2 diabetes mellitus (T2DM) is a chronic metabolic disease causing an increase in morbidity and mortality [1]. Globally, around 400 million people have diabetes, and it is expected that by 2040, diabetes incidence will increase to 640 million [2]. In 2019, the Global Burden of Diseases Study reported that diabetes is the direct cause of 1.5 million deaths [3]. The World Health Organization (WHO) reported that Saudi Arabia ranks second in the middle east with a high incidence of type 2 diabetes mellitus [4]. Consequently, type 2 diabetes mellitus is associated with many other chronic illnesses, including kidney failure (>50%), cardiovascular diseases (CVD) (70%), vision loss, and other related diseases [3]. The major complication of type 2 diabetes mellitus is the progression of macrovascular and microvascular complications [5]. Hence, type 2 diabetes mellitus patients are more susceptible to atherosclerosis and stroke than people without type 2 diabetes mellitus disease [1,6]. One of the significant risk factors for type 2 diabetes mellitus is obesity and its associated dyslipidemia and insulin resistance [7,8].

Dyslipidemia is an abnormal circulating blood level of lipids, including cholesterol, low/very low-density lipoprotein, high-density lipoprotein, and triglycerides [8]. These can lead to the development of atherosclerosis and CVD [8]. The increase in insulin levels leads to the rise of circulating free fatty acids, which is the main defect of lipid profiles in diabetic patients [6]. Many therapeutic strategies for diabetic patients have been used in the last decades. These strategies include lifestyle modification, weight loss, physical activity, and non-insulin and insulin medications [9,10]. The therapeutic management of type 2 diabetes mellitus includes metformin, sulfonylureas, insulin therapy, and thiazolidinediones [10]. Additionally, a recent development involves a new therapy that targets diabetic dyslipidemia, such as glucagon-like peptide receptor agonists (GLP-1RA) [6,10].

GLP-1 is a peptide hormone related to the glucagon superfamily [11] and shares a significant amino acid sequence with glucagon [12]. The glucagon superfamily peptides are secreted from the small intestine, pancreas, brain, and peripheral nerves [12]. They can act as hormones or neurotransmitters [6]. The proglucagon gene encodes the glucagon superfamily peptides, which generate a single precursor protein. It undergoes co-translation and post-translation processing to rise to different active peptides [12]. GLP-1 is one of these peptides secreted from the small intestine [9]. GLP-1 can also be expressed in tissues such as the endocrine pancreas, lower brain regions, and the large intestine [13]. GLP-1 secretion into the bloodstream is stimulated by food consumption or glucose; the active GLP-1 peptide disappears within 3 h [14]. GLP-1 acts as one of the primary regulators of blood glucose levels and stimulates insulin secretion in response to hyperglycemia [15]. GLP-1 acts as an incretin hormone by augmenting insulin release and inhibiting glucagon secretion, thereby regulating the postprandial glucose level [16]. A study by Drucker et al. found that GLP-1 showed an effective glucose-dependent insulin secretion in normal and diabetic animals and human tissues [9]. These findings, followed rapidly by another study, demonstrated that GLP-1 inhibited glucagon secretion, food intake, and gastric emptying [9].

Furthermore, GLP-1 controls intestinal motility and decreases gastric motility [17]. It also has an effect of satiety, which may be attributed to its effect on the gut, but it also has a direct effect on the hypothalamic feeding centres [17]. Consequently, these observations have led to diabetes therapy development in the form of GLP-1 receptor (GLP-1R) agonists for the treatment of type 2 diabetes mellitus and, subsequently, for obesity [9].

The establishment of GLP-1 receptor agnostic (GLP-1 RA) therapy has been used to improve insulin sensitivity in obese and diabetic patients [11]. GLP-1RA inhibits glucagon secretion from the pancreatic alpha cells when blood sugar levels are high and can decrease pancreatic beta cell apoptosis in proliferation [18,19,20]. Moreover, GLP-1RA showed an effect on satiety improvement and body weight loss. Thus, it can also be used for weight loss and dyslipidemia therapy [21]. Therefore, the GLP-1RA can be suitable for reducing plasma glucose in type 2 diabetes mellitus patients with dyslipidemia [11].

GLP-1RAs augment glucose-stimulated insulin secretion (GSIS) and suppress glucagon secretion at hyperglycemic or euglycemic conditions. Moreover, GLP-1RAs regulate cardiovascular and endothelial cell functions through an anti-inflammatory, antioxidant, and vasodilator effect on endothelial cells [22,23]. Thus, the reported benefits of GLP-1RA use in patients with CVD include a reduction in the risk of cardiovascular events [24]

Until now, there is no reported data regarding the long-term effects of the therapeutic use of GLP-1RA on diabetes associated with dyslipidemia in Saudi Arabia. The current study aims to retrospectively assess the possible association of the long-term use of GLP-1RA effect in Saudi type 2 diabetes mellitus patients with Hb A1c and dyslipidemia.

## 2. Experimental Design

An observational retrospective study included patients with type 2 diabetes mellitus (*n* = 72) adults older than 18 years who had been prescribed their first-time GLP-1 RA, such as liraglutide and/or semaglutide treatments. The study was conducted between January 2019 and December 2021 at King Abdulaziz University Hospital (KAUH), Jeddah, Saudi Arabia. Type 2 diabetes mellitus patients were following up regularly with their diabetology clinic at KAUH. The Research Ethics Committee at the Faculty of Medicine—King Abdulaziz University, Jeddah, Saudi Arabia, approved the study, reference no. 231-22. The electronic clinical database for patients and follow-up records were all obtained from the KAUH phoenix database system (phoenix). The demographic characteristics database for patients was collected at the starting date of GLP-1RA treatments and included the following: age, nationality, gender, height, history of statin medication, and comorbidities. The therapeutic protocol of liraglutide and semaglutide was as follows: liraglutide 0.6 mg subcutaneous injection daily for one week; weekly increase until it reached 3 mg. For Semaglutide, 0.25 mg subcutaneously once a week for four weeks, then progressively increased until it reached 1 mg; six patients received combined starting with liraglutide and shifted to semaglutide [25].

Additionally, patients selected for the study had pre- and post-medication records. The BMI, weight, and biochemical parameters were recorded twice over an estimated six-month period based on the pre-and post-index of each. The biochemical parameters included are cholesterol, triglycerides (TG), low-density lipoproteins (LDL), high-density lipoprotein (HDL), aspartate aminotransferase (AST), alanine aminotransferase (ALT), high sensitivity c-reactive protein (hs-CRP) and Hb-A1C. The low-density lipoprotein (VLDL) values were obtained using lipid panel values by Friedewald’s equation calculation [26].

The inclusion criteria included subjects with type II DM with dyslipidemia who received GLP-1RA for six months or more. The type of dyslipidemia (hypertriglyceridemia, hypercholesterolemia, and disturbed LDL/HDL ratio) in those patients is of the nonfamilial and non-genetic types.

**Figure 1:**
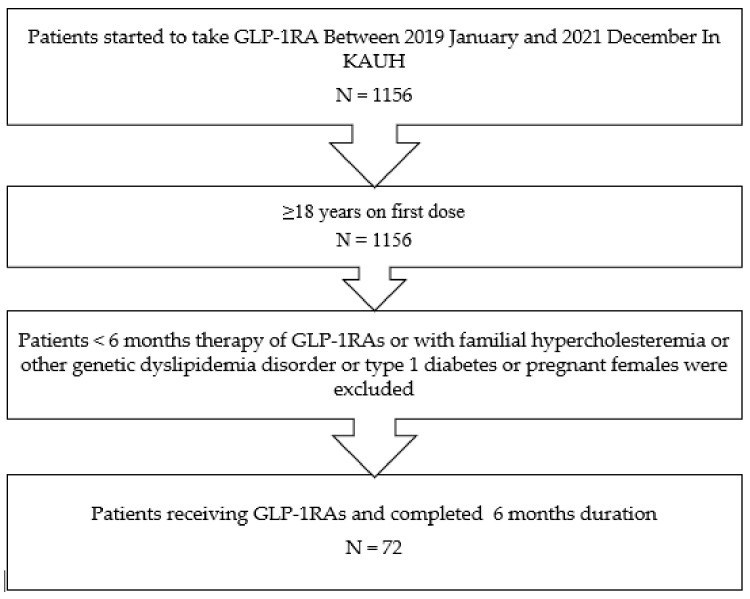
A flow chart presenting the sequence of data collection of patient records according to the inclusion and exclusion criteria of the study. One thousand one hundred fifty-six diabetic patients’ records were examined; patients over 18 years old who completed at least six months of GLP-1RA therapy and with clinical records from both pre- and post-GLP-1RA treatment were included in the study.

Exclusion criteria included patients with less than six months of GLP-1RA use, patients with familial hypercholesteremia or other genetic dyslipidemia, type 1 diabetes, patients with abnormal thyroid function tests, and pregnant females, as well as patients who had undergone bariatric surgery or taken weight-loss medications, such as orlistat. A flowchart of the selection criteria, including inclusion and exclusion criteria, is given in Figure 1.

### Statistical Analysis

Data analysis was performed using IBM SPSS statistics version 23.0 for Windows. Baseline characteristics were expressed as the mean and standard error of the mean (SEM). An independent *t*-test analysis was used to compare factors between the two groups (statin vs. no statin), while paired *t*-test analysis was used to compare baseline and six months. The Pearson correlation coefficient (r) was used to study the correlation between the dose of GLP-1 receptor agonist and different parameters.

## 3. Results

A summary of demographic and anthropometrics results presents in Table 1. In total, 72 diabetic patient records (28 male and 44 female) were analyzed. The average age was 55 years old. The average height was 171 cm in males and 156 cm in females, and the average BMI was 36.24 Kg/m^2^ in males and 38 Kg/m^2^ in females. Furthermore, 46 (64%) of the patients were Saudis. In total, 29 patients were also diagnosed with hypertension and 8 with heart failure. Moreover, 63 patients (22 males and 41 females) were prescribed cholesterol-lowering medication in the form of high-intensity statins, rosuvastatin (36), and atorvastatin (27). Accordingly, the study sample was divided into two groups, group 1 patients who received statin medication (*n* = 63) and group 2 patients without statin medication (*n* = 9). The patient diabetic drug history showed that 39 (54%) were taking insulin, 76% were taking metformin, 18% were taking gliclazide, 8% were taking repaglinide, and 2% were taking glimepiride; their distributions between the statin and the non-statin group is presented in Table 2.

**Figure 2:**
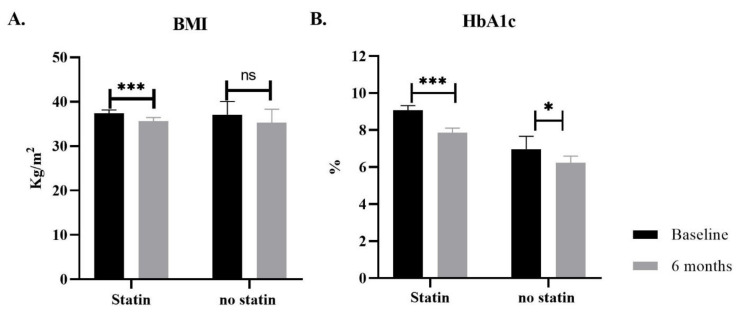
Bar graph representing the effect of GLP-1RA treatment on BMI and HbA1c in diabetic patients. Two groups of diabetic patients received GLP-1RA treatment for six months. Alongside their regular diabetes management medication, the first group of patients was on an antihyperlipidemic drug (Statin), and the 2nd group was not. (**A**) The effect on BMI significantly decreased only in the group taking statins. (**B**) GLP-1RA treatment has an effect of HbA1c on both groups, the effect more significant in the statin group. Data presented as mean ± SEM, *** *p*-value ≤ 0.001, * *p*-value ≤ 0.05., ns: non-significant.

Patients received different doses of GLP-1RA as subcutaneous injections for at least six months. Other parameters and biochemical tests were recorded at baseline (before receiving GLP-1RA) and during follow-up (after six months). The most significant finding can be seen in BMI and HbA1c, detailed in Table 3 and Figure 2. The BMI values were significantly changed from 37.3 kg/m^2^ to 35.6 kg/m^2^ with a *p*-value < 0.001 in the statin group. However, no statistically significant differences showed in the no-statin group.

Furthermore, HbA1c significantly decreased in both groups, from 9.2% to 7.9% *p*-value < 0.001 in the statin group and from 6.9% to 5.8% *p*-value < 0.03 in the no statin group.

Lipid profiles were recorded at baseline and after 6 months of GLP-1RA treatment. Some minor decreases could be seen in TG and VLDL levels in the no-statin group from 2.1 mmol/L to 1.7 mmol/L and 38.7 mg/dL to 30.37 mg/dL, respectively, but the changes were not statistically significant. On the other hand, GLP-1RA treatments showed no remarkable changes in cholesterol, HDL or LDL levels, as seen in Table 4.

Results for liver enzymes and hs-CRP levels are presented in Table 5. ALT levels were decreased in both groups from 25.1 to 22.9 U\L in the statin group, with a *p*-value of 0.09 trending toward significance, and from 27.2 to 25.2 U\L in the no statin group. AST levels decreased only in the no statin group from 25.2 to 19.4 U\L and a *p*-value of 0.011. However, no apparent changes in the hs-CRP levels were found.

Finally, patients received different doses of GLP-1 receptors agonist ranging from 0.25–3 mg\week. Thus, the correlation analysis was conducted to examine the relationship between the dosage and changes observed during the six months in any parameter shown in Table 6. There was a negative correlation between the dose and changes in LDL r = −0.326, with a *p*-value of 0.024. Conversely, the changes in cholesterol also negatively correlate with the dose, with the *p*-value trending toward a significant 0.08.

## 4. Discussion

GLP-1RAs have been used as an important established therapy for diabetic patients throughout the last decade. Other diabetic therapies, including metformin, are still the standard first-line treatment for type 2 diabetes mellitus, according to the American Diabetes Association guidelines. Nevertheless, GLP-1RAs, in addition to type 2 diabetes mellitus patients therapy, are also considered for patients with metformin intolerance and HbA1c levels higher than 1.5% of the normal target range [27,28]. In the current study, we investigated the association between the long-term use of GLP-1RAs with glycemic control and lipid profile in diabetic patients at King Abdulaziz University Hospital in Jeddah. Alanazi et al. group studied the effect of GLP-1RAs on body weight, body mass index, and HbA1c before and after the six-month use of GLP-1RA in patients with type II diabetes mellitus [29]. Their results revealed a significant reduction in BMI with no significant difference in HbA1c [29]. In the present study, we revealed a significant reduction in BMI in GLP-1RA-treated subjects who were treated with statin (lipid-lowering drugs). This is consistent with other previous studies that showed the effect of GLP-1RAs on reducing body weight [29,30,31,32,33,34,35]. Thus, GLP-1RAs lower the appetite and, consequently, decrease food intake by increasing satiety and a feeling of stomach fullness [25]. It also decreases gastrointestinal motility and reduces calorie intake [9,23]. This action occurs via the GLP-1RAs mechanism which involves stimulating insulin secretion and reducing glucagon secretion [9,22]. Furthermore, GLP-1RAs have an anorectic effect as they activate GLP-1Rs in the arcuate nucleus of the brain. Hence, GLP-1RAs act centrally on the brain–adipcyte axis. This mechanism could explain the anti-obesity effect of GLP-1RAs [25,36].

Regarding the relationship between GLP-1RAs and glycemic control, our findings show a significant decrease in HbA1c levels. It is found in the GLP-1RAs treated subjects with and without lipids-lowering drug therapy. This finding proves its role in managing glycemic parameters, consistent with previous studies [37,38,39,40]. Thus, it has been reported that GLP-1RAs enhance beta cell functions, which improve insulin sensitivity and reduce glucagon secretion to the lowest basal level [37]. Therefore, GLP-1RAs are effectively unique in lowering glycemic levels and reducing body weight. They also promote a decrease in glucosuria levels [25].

An increase in Homeostasis Model Assessment-2 B (HOMA2-B) indices and a decrease in proinsulin levels, proinsulin/C-peptide ratios, and proinsulin/insulin ratios were also reported to be a direct effect of GLP-1RAs [41,42]. Moreover, GLP-1RAs therapy can be associated with increased adiponectin gene expression and serum adiponectin level [43]. Adiponectin enhances the GLUT4-mediated glucose uptake in the white adipocytes, decreases hepatic lipid accumulation, and minimizes visceral fat depots [44]. Furthermore, GLP-1RAs improve endothelial cell function through their anti-inflammatory effect [45]. The anti-inflammatory effects of GLP-1RAs on macrophages protect against the development of atherosclerosis.

Moreover, it is confirmed that GLP-1RAs produce a protective effect against hepatotoxicity and hepatic steatosis [46]. A study by Ohki et al. observed that GLP-1RA directly decreases liver fibrosis and steatosis in vivo. This effect is the principle of potential GLP-1RA therapeutic use for nonalcoholic fatty liver diseases (NAFLD) [47,48]. Consequently, the relationship between GLP-1RAs and AST serum level was also reported by Hartman et al. [46]. One of the apparent observations of the current study is the decrease in AST serum level, with no change observed regarding ALT. The improvement of one of the vital liver functions could be explained by the anti-inflammatory effect of GLP-1RAs [45] and the protection against lipotoxicity [44]. However, the direct effect of GLP-1RAs on reducing liver enzyme levels is not fully understood, requiring further investigation [47].

Surprisingly, the results of the current study did not demonstrate an association between GLP-1RAs treatment and lipid profile. No consistent relationship was observed with individual lipid profile components, including total cholesterol, triglyceride, LDL-C, HDL-C, and VLDL levels in subjects, either with or without lipids-lowering drugs(statin) therapy. Only a significant negative correlation was observed between GLP-1RAs dose and LDL-C level variations between pre-and post-treatment with GLP-1RAs. These findings were in accordance with the previous reports that showed an indirect, no direct association of GLP-1RA treatment effect with lipid profile levels [47,48,49]. However, Sun et al. reported a negative association between GLP-1RA monotherapy without statin therapy and LDL-C, total cholesterol, and triglycerides but no considerable improvement in HDL-C level [41]. In addition, Hasegawa et al. reported that it reduced LDL-C in patients with type II diabetes mellitus treated with statins in their study in Japan [50].

Collectively, the present study is one of few studies that assess the long-term association effect of GLP-1RAs therapy in patients with type II diabetes Mellitus in Saudi Arabia. In 2020, Alanazi and Ghoraba retrospectively investigated the association between the GLP-1RA treatment of type II diabetes mellitus and body mass index [21]. They concluded that liraglutide (one of the most widely used GLP-1RA therapeutic products in the King Abdulaziz University Hospital) has a crucial effect on weight loss and reduction in body mass index. One of the recent studies investigated the sodium-glucose cotransporter-2 inhibitors (SGLT2i) as a co-therapy with GLP-1 RAs for treating type II diabetes mellitus [51]. Both studies did not examine the potential effect of GLP-1 RA treatment on the serum lipid profile [29,51].

Our findings showed GLP-1RAs treatments are associated with weight reduction and improved glycemic control for patients of type II diabetes mellitus but have no direct effect on lipid profile. However, the current study has a few limitations, including data extracted from a single medical centre (King Abdulaziz University Hospital) and the limited sample number. Therefore, obtaining data from multiple medical centres could improve the findings regarding the effects of GLP-1RA therapy, with or without other lipid-lowering drugs. The limited number of the analyzed data after the exclusion of the results did not meet the inclusion criteria of our study. Moreover, the difficulty in obtaining access to the selected cases’ personal, other medical, and therapeutic data. A degree of individual compliance is necessary in order to use the drug. Further randomized multicentric studies in Saudi Arabia are needed to confirm the results of the current study and to fairly assess the drug’s efficacy among GLP-1RA receivers for either the weight reduction or treatment of diabetes. Additionally, investigating other possible favorable or unfavorable effects is important.

## 5. Conclusions

This retrospective study concludes that GLP-1RAs treatment is associated with weight reduction and better glycemic control in type 2 diabetes within a six-month period. However, no direct association was observed regarding its relation to serum lipid profile except a negative correlation between GLP-1RAs with LDL-C.

## Figures and Tables

**Table 1:** Demographic, anthropometric, and clinical characteristics of the studied groups.

	Male *n* = 28		Female *n* = 44	
	Mean	SEM	Mean	SEM
**Age** **(years)**	55	2	55	1
**Height** **(cm)**	171.86	1.60	156.35	1.12
**BMI (kg\m^2^)**	36.24	1.05	38.00	1.07
	**Count**	**%**	**Count**	**%**
**Nationality**				
Saudi	20	43.5%	26	56.5%
Non-Saudi	8	30.8%	18	69.2%
**Hypertension**				
Yes	11	36.7%	19	63.3%
No	17	40.5%	25	59.5%
**Heart Failure**				
Yes	2	25.0%	6	75.0%
No	25	39.7%	38	60.3%
**Statin**				
Yes	22	34.9%	41	65.1%
No	6	66.7%	3	33.3%

**Table 2:** Different management of type 2 diabetes mellitus patients in the study groups.

	Statin		No Statin	
	Count	%	Count	%
**Insulin**				
Yes	37	94.9%	2	5.1%
No	26	78.1%	7	21.9%
**Metformin**				
Yes	49	94.2%	3	5.8%
No	14	70.0%	6	30.0%
**Gliclazide**				
Yes	11	84.6%	2	15.4%
No	52	88.1%	7	11.9%
**Repaglinide**				
Yes	6	100.0%	0	0.0%
No	57	86.4%	9	13.6%
**Glimepiride**				
Yes	2	100.0%	0	0.0%
No	61	87.1%	9	12.9%

**Table 3:** Changes of BMI and HbA1c from the baseline in type 2 diabetes mellitus subjects treated with GLP-1RAs.

	*n*	Baseline	Month 6	The Difference at Month 6	*p*-Value	
					Baseline vs. 6 Months ^a^	Between Groups, Statins vs. No Statins ^b^
**BMI(kg\m^2^)**						0.449
Statin	59	37.3 ± 0.84	35.63 ± 0.8	−1.68 ± 0.4	**<0.001**	
No Statin	7	36.04 ± 3.23	35.26 ± 3.07	−0.78 ± 0.58	0.228	
**HbA1c (%)**						0.736
Statin	53	9.24 ± 0.27	7.94 ± 0.24	−1.3 ± 0.24	**<0.001**	
No Statin	7	6.94 ± 0.79	5.87 ± 0.44	−1.07 ± 0.39	**0.032**	

BMI: body mass index, HbA1c: hemoglobin A1c, ^a^ paired test, ^b^ independent *t*-test. Bold and underlined values indicate statistical significance.

**Table 4:** Changes of lipid profile levels from the baseline to 6 months duration of GLP-1RA treatment in type 2 diabetes mellitus subjects.

	*n*	Baseline	Month 6	Difference at Month 6	*p*-Value	
					Baseline vs. 6 Months ^a^	Between Groups, Statins vs. No Statins ^b^
**Cholesterol(mmol/L)**						0.944
Statin	52	4.04 ± 0.14	3.94 ± 0.12	−0.1 ± 0.13	0.441	
No Statin	7	4.49 ± 0.31	4.36 ± 0.26	−0.13 ± 0.19	0.515	
**TG (mmol/L)**						0.192
Statin	52	1.72 ± 0.11	1.73 ± 0.16	0 ± 0.13	0.972	
No Statin	7	2.19 ± 0.43	1.71 ± 0.19	−0.47 ± 0.31	0.177	
**HDL (mmol/L)**						0.828
Statin	19	1.22 ± 0.08	1.49 ± 0.37	0.28 ± 0.38	0.479	
No Statin	3	1.03 ± 0.17	1.09 ± 0.18	0.06 ± 0.07	0.501	
**LDL (mmol/L)**						0.665
Statin	44	2.6 ± 0.16	2.45 ± 0.15	−0.15 ± 0.15	0.322	
No Statin	4	2.63 ± 0.34	2.7 ± 0.51	0.07 ± 0.25	0.794	
**VLDL (mg/dL)**						0.192
Statin	52	30.54 ± 1.93	30.62 ± 2.83	0.08 ± 2.22	0.972	
No Statin	7	38.72 ± 7.62	30.37 ± 3.36	−8.35 ± 5.46	0.177	

TG: triglyceride, HDL, high-density lipoprotein, LDL: low-density lipoprotein, VLDL: very low-density lipoprotein, ^a^ paired test, ^b^ independent *t*-test.

**Table 5:** Changes of ALT, AST, and hs-CRP levels from the baseline to 6 months in type 2 diabetes mellitus subjects treated with GLP-1RAs.

	*n*	Baseline	Month 6	Difference at Month 6	*p*-Value	
					Baseline vs. 6 Months ^a^	Between Groups, Statins vs. no Statins ^b^
**ALT(U/L)**						0.965
Statin	39	25.1 ± 2	22.92 ± 1.57	−2.18 ± 1.25	0.09	
No Statin	7	27.29 ± 6.38	25.29 ± 5.18	−2 ± 6.83	0.780	
**AST(U/L)**						**0.012**
Statin	39	20.62 ± 1.32	22.28 ± 1.37	1.67 ± 1.18	0.166	
No Statin	7	25.29 ± 3.46	19.43 ± 2.1	−5.86 ± 1.61	**0.011**	
**hs-CRP (mg/L)**						0.968
Statin	6	11.65 ± 8.35	12.24 ± 5.53	0.59 ± 9.2	0.951	
No Statin	1	3.3	2.86	−0.44 ± .		

ALT; alanine aminotransferase, AST: aspartate aminotransferase, hs-CRP: high sensitivity C-reactive protein, ^a^ paired test, ^b^ independent *t*-test. Bold and underlined values indicate statistical significance.

**Table 6:** Correlation between GLP-1 agonist dose and change in clinical parameters.

	*n*	r	*p*-Value
**ΔBMI**	66	−0.146	0.241
**ΔChol**	59	−0.228	0.083
**ΔTG**	59	−0.119	0.371
**ΔHDL**	22	0.104	0.645
**ΔLDL**	48	−0.326	**0.024**
**ΔVLDL**	59	−0.12	0.367
**ΔALT**	46	0.224	0.134
**ΔAST**	46	−0.014	0.927
**ΔHbA1C**	60	0.083	0.529
**Δhs-CRP**	7	0.267	0.562

Bold and underlined values indicate statistical significance.

## Data Availability

The datasets analyzed for this study can be found at https://drive.google.com/file/d/1r4o21-vgd8Ret7PgMZNFYE9lMWpmzVVt/view?usp=share_link (Last accessed on 1 February 2023).

## References

[1] Pyörälä K., Laakso M., Uusitupa M. (1987). Diabetes and atherosclerosis: An epidemiologic view. Diabetes/Metab. Res. Rev..

[2] Swami O.C., Sharma S.K., Panneerselvam A., Singh K.P., Parmar G., Gadge P. (2016). Teneligliptin in management of type 2 diabetes mellitus. Diabetes Metab. Syndr. Obes. Targets Ther..

[3] (2020). Global Burden of Disease Study 2019; Latest GBD Results: 2019. https://www.healthdata.org/gbd/gbd-2019-resources.

[4] Al Dawish M.A., Robert A.A., Braham R., Al Hayek A.A., Al Saeed A., Ahmed R.A., Al Sabaan F.S. (2016). Diabetes Mellitus in Saudi Arabia: A Review of the Recent Literature. Curr. Diabetes Rev..

[5] Almeda-Valdes P., Cuevas-Ramos D., Mehta R., Gomez-Perez F., Aguilar-Salinas C. (2010). UKPDS Risk Engine, Decode and Diabetes PHD Models for the Estimation of Cardiovascular Risk in Patients with Diabetes. Curr. Diabetes Rev..

[6] Patel V., Joharapurkar A., Shah G., Jain M. (2014). Effect of GLP-1 Based Therapies on Diabetic Dyslipidemia. Curr. Diabetes Rev..

[7] Hemmer A., Maiter D., Buysschaert M., Preumont V. (2018). Long-term effects of GLP-1 receptor agonists in type 2 diabetic patients: A retrospective real-life study in 131 patients. Diabetes Metab. Syndr. Clin. Res. Rev..

[8] Ormazabal V., Nair S., Elfeky O., Aguayo C., Salomon C., Zuñiga F.A. (2018). Association between insulin resistance and the development of cardiovascular disease. Cardiovasc. Diabetol..

[9] Drucker D.J. (2018). Mechanisms of Action and Therapeutic Application of Glucagon-like Peptide-1. Cell Metab..

[10] Chaudhury A., Duvoor C., Reddy Dendi V.S., Kraleti S., Chada A., Ravilla R., Marco A., Shekhawat N.S., Montales M.T., Kuriakose K. (2017). Clinical Review of Antidiabetic Drugs: Implications for Type 2 Diabetes Mellitus Management. Front. Endocrinol..

[11] Nauck M.A., Meier J.J. (2016). GLP-1 receptor agonists and SGLT2 inhibitors: A couple at last?. Lancet Diabetes Endocrinol..

[12] Seon M.J., Hwang S.Y., Son Y., Song J., Kim O.Y. (2021). Circulating GLP-1 Levels as a Potential Indicator of Metabolic Syndrome Risk in Adult Women. Nutrients.

[13] Lee C.-H., Jeon S.J., Cho K.S., Moon E., Sapkota A., Jun H.S., Ryu J.H., Choi J.W. (2017). Activation of Glucagon-Like Peptide-1 Receptor Promotes Neuroprotection in Experimental Autoimmune Encephalomyelitis by Reducing Neuroinflammatory Responses. Mol. Neurobiol..

[14] D’Alessio D. (2016). IsGLP-1 a hormone: Whether and When?. J. Diabetes Investig..

[15] Sandoval D.A., D’Alessio D.A. (2015). Physiology of Proglucagon Peptides: Role of Glucagon and GLP-1 in Health and Disease. Physiol. Rev..

[16] Holst J.J. (2007). The Physiology of Glucagon-like Peptide 1. Physiol. Rev..

[17] MacDonald P.E., El-Kholy W., Riedel M.J., Salapatek A.M.F., Light P.E., Wheeler M.B. (2002). The Multiple Actions of GLP-1 on the Process of Glucose-Stimulated Insulin Secretion. Diabetes.

[18] Garber A.J. (2011). Long-Acting Glucagon-Like Peptide 1 Receptor Agonists. Diabetes Care.

[19] Gallwitz B. (2011). GLP-1 Agonists and Dipeptidyl-Peptidase IV Inhibitors. Diabetes—Perspectives in Drug Therapy.

[20] Okerson T., Chilton R.J. (2010). The Cardiovascular Effects of GLP-1 Receptor Agonists. Cardiovasc. Ther..

[21] Cena H., Calder P.C. (2020). Defining a Healthy Diet: Evidence for the Role of Contemporary Dietary Patterns in Health and Disease. Nutrients.

[22] Andreadi A., Bellia A., Di Daniele N., Meloni M., Lauro R., Della-Morte D., Lauro D. (2021). The molecular link between oxidative stress, insulin resistance, and type 2 diabetes: A target for new therapies against cardiovascular diseases. Curr. Opin. Pharmacol..

[23] Bray J.J.H., Foster-Davies H., Salem A., Hoole A.L., Obaid D.R., Halcox J.P.J., Stephens J.W. (2021). Glucagon-like peptide-1 receptor agonists improve biomarkers of inflammation and oxidative stress: A systematic review and meta-analysis of randomised controlled trials. Diabetes Obes. Metab..

[24] Bethel M.A., Patel R.A., Merrill P., Lokhnygina Y., Buse J.B., Mentz R.J., Pagidipati N.J., Chan J.C., Gustavson S.M., Iqbal N. (2018). Cardiovascular outcomes with glucagon-like peptide-1 receptor agonists in patients with type 2 diabetes: A meta-analysis. Lancet Diabetes Endocrinol..

[25] Nauck M.A., Quast D.R., Wefers J., Meier J.J. (2020). GLP-1 receptor agonists in the treatment of type 2 diabetes—State-of-the-art. Mol. Metab..

[26] Wilson P.W., Zech L.A., Gregg R.E., Schaefer E.J., Hoeg J.M., Sprecher D.L., Brewer H. (1985). Estimation of VLDL cholesterol in hyperlipidemia. Clin. Chim. Acta.

[27] Hunt B., Malkin S.J.P., Moes R.G.J., Huisman E.L., Vandebrouck T., Wolffenbuttel B.H.R. (2019). Once-weekly semaglutide for patients with type 2 diabetes: A cost-effectiveness analysis in the Netherlands. BMJ Open Diabetes Res. Care.

[28] Burcelin R., Gourdy P. (2016). Harnessing glucagon-like peptide-1 receptor agonists for the pharmacological treatment of overweight and obesity. Obes. Rev..

[29] Alanazi N.K., Ghoraba M.A. (2020). Effect of Glucagon-like peptide-1 agonist (liriglutide) on weight and glycemic control among adults with type 2 diabetes mellitus attending primary care center at security forces hospital in Riyadh, Saudi Arabia. J. Fam. Med. Prim. Care.

[30] Cena H., Chiovato L., Nappi R.E. (2020). Obesity, Polycystic Ovary Syndrome, and Infertility: A New Avenue for GLP-1 Receptor Agonists. J. Clin. Endocrinol. Metab..

[31] Sattar N., Lee M.M.Y., Kristensen S.L., Branch K.R.H., Del Prato S., Khurmi N.S., Lam C.S.P., Lopes R.D., McMurray J.J.V., Pratley R.E. (2021). Cardiovascular, mortality, and kidney outcomes with GLP-1 receptor agonists in patients with type 2 diabetes: A systematic review and meta-analysis of randomised trials. Lancet Diabetes Endocrinol..

[32] Ryan P.M., Seltzer S., Hayward N.E., Rodriguez D.A., Sless R.T., Hawkes C.P. (2021). Safety and Efficacy of Glucagon-Like Peptide-1 Receptor Agonists in Children and Adolescents with Obesity: A Meta-Analysis. J. Pediatr..

[33] Guo N., Sun J., Chen H., Zhang H., Zhang Z., Cai D. (2013). Liraglutide prevents diabetes progression in prediabetic OLETF rats. Endocr. J..

[34] Yousef C.C., Thomas A., Al Matar M., Ghandoura L., Aldossary I., Almuhanna S.M., Alhussain F., AL Bisher F.B., Aljohani R.M., Balubaid A.N. (2021). Liraglutide effects on glycemic control and weight in patients with type 2 diabetes Mellitus: A real-world, observational study and brief narrative review. Diabetes Res. Clin. Pract..

[35] Alrowais S.S. (2021). Relationship between exposure to Liraglutide and weight loss: A cross-sectional study in Riyadh, Saudi Arabia. Int. J. Clin. Exp. Med..

[36] Guo X., Zhou Z., Lyu X., Xu H., Zhu H., Pan H., Wang L., Yang H., Gong F. (2022). The Antiobesity Effect and Safety of GLP-1 Receptor Agonist in Overweight/Obese Patients Without Diabetes: A Systematic Review and Meta-Analysis. Horm. Metab. Res..

[37] Thomas M.K., Nikooienejad A., Bray R., Cui X., Wilson J., Duffin K., Milicevic Z., Haupt A., Robins D.A. (2020). Dual GIP and GLP-1 Receptor Agonist Tirzepatide Improves Beta-cell Function and Insulin Sensitivity in Type 2 Diabetes. J. Clin. Endocrinol. Metab..

[38] Bethel M.A., Diaz R., Castellana N., Bhattacharya I., Gerstein H.C., Lakshmanan M.C. (2020). HbA1c Change and Diabetic Retinopathy During GLP-1 Receptor Agonist Cardiovascular Outcome Trials: A Meta-analysis and Meta-regression. Diabetes Care.

[39] Ryan D.H. (2021). Next Generation Antiobesity Medications: Setmelanotide, Semaglutide, Tirzepatide and Bimagrumab: What do They Mean for Clinical Practice?. J. Obes. Metab. Syndr..

[40] Karagiannis T., Avgerinos I., Liakos A., Del Prato S., Matthews D.R., Tsapas A., Bekiari E. (2022). Management of type 2 diabetes with the dual GIP/GLP-1 receptor agonist tirzepatide: A systematic review and meta-analysis. Diabetologia.

[41] Sun F., Wu S., Wang J., Guo S., Chai S., Yang Z., Li L., Zhang Y., Ji L., Zhan S. (2015). Effect of Glucagon-like Peptide-1 Receptor Agonists on Lipid Profiles Among Type 2 Diabetes: A Systematic Review and Network Meta-analysis. Clin. Ther..

[42] Sun J., Cui J., He Q., Chen Z., Arvan P., Liu M. (2015). Proinsulin misfolding and endoplasmic reticulum stress during the development and progression of diabetes☆. Mol. Asp. Med..

[43] Ceperuelo-Mallafré V., Duran X., Pachón G., Roche K., Garrido-Sánchez L., Vilarrasa N., Tinahones F.J., Vicente V., Pujol J., Vendrell J. (2014). Disruption of GIP/GIPR Axis in Human Adipose Tissue Is Linked to Obesity and Insulin Resistance. J. Clin. Endocrinol. Metab..

[44] Stern J.H., Rutkowski J.M., Scherer P.E. (2016). Adiponectin, Leptin, and Fatty Acids in the Maintenance of Metabolic Homeostasis through Adipose Tissue Crosstalk. Cell Metab..

[45] Ma X., Liu Z., Ilyas I., Little P.J., Kamato D., Sahebka A., Chen Z., Luo S., Zheng X., Weng J. (2021). GLP-1 receptor agonists (GLP-1RAs): Cardiovascular actions and therapeutic potential. Int. J. Biol. Sci..

[46] Hartman M.L., Sanyal A.J., Loomba R., Wilson J.M., Nikooienejad A., Bray R., Karanikas C.A., Duffin K.L., Robins D.A., Haupt A. (2020). Effects of Novel Dual GIP and GLP-1 Receptor Agonist Tirzepatide on Biomarkers of Nonalcoholic Steatohepatitis in Patients With Type 2 Diabetes. Diabetes Care.

[47] Ohki T., Isogawa A., Iwamoto M., Ohsugi M., Yoshida H., Toda N., Tagawa K., Omata M., Koike K. (2012). The Effectiveness of Liraglutide in Nonalcoholic Fatty Liver Disease Patients with Type 2 Diabetes Mellitus Compared to Sitagliptin and Pioglitazone. Sci. World J..

[48] Rezaei S., Tabrizi R., Nowrouzi-Sohrabi P., Jalali M., Atkin S.L., Al-Rasadi K., Jamialahmadi T., Sahebkar A. (2021). GLP-1 Receptor Agonist Effects on Lipid and Liver Profiles in Patients with Nonalcoholic Fatty Liver Disease: Systematic Review and Meta-Analysis. Can. J. Gastroenterol. Hepatol..

[49] Aoki K., Kamiyama H., Takihata M., Taguri M., Shibata E., Shinoda K., Yoshii T., Nakajima S., Terauchi Y. (2020). Effect of liraglutide on lipids in patients with type 2 diabetes: A pilot study. Endocr. J..

[50] Hasegawa Y., Hori M., Nakagami T., Harada-Shiba M., Uchigata Y. (2018). Glucagon-like peptide-1 receptor agonists reduced the low-density lipoprotein cholesterol in Japanese patients with type 2 diabetes mellitus treated with statins. J. Clin. Lipidol..

[51] Korayem G.B., Alshaya O.A., Alghamdi A.A., Alanazi S.S., Almutib R.T., Alsaileek M., Alrashidi A., Aldosari N., Bin Sheraim N., Al Yami M.S. (2022). The prescribing pattern of sodium-glucose cotransporter-2 inhibitors and glucagon-like peptide-1 receptor agonists in patient with type two diabetes mellitus: A two-center retrospective cross-sectional study. Front. Public Health.

